# Supplementary figures and images for: Thymol, a Monoterpenoid within Polymeric Iodophor Formulations and Their Antimicrobial Activities
Source: Int J Mol Sci. 2024 May 1;25(9):4949. doi: 10.3390/ijms25094949 (PMC11084924; doi:10.3390/ijms25094949)

EDS Layered Image 5

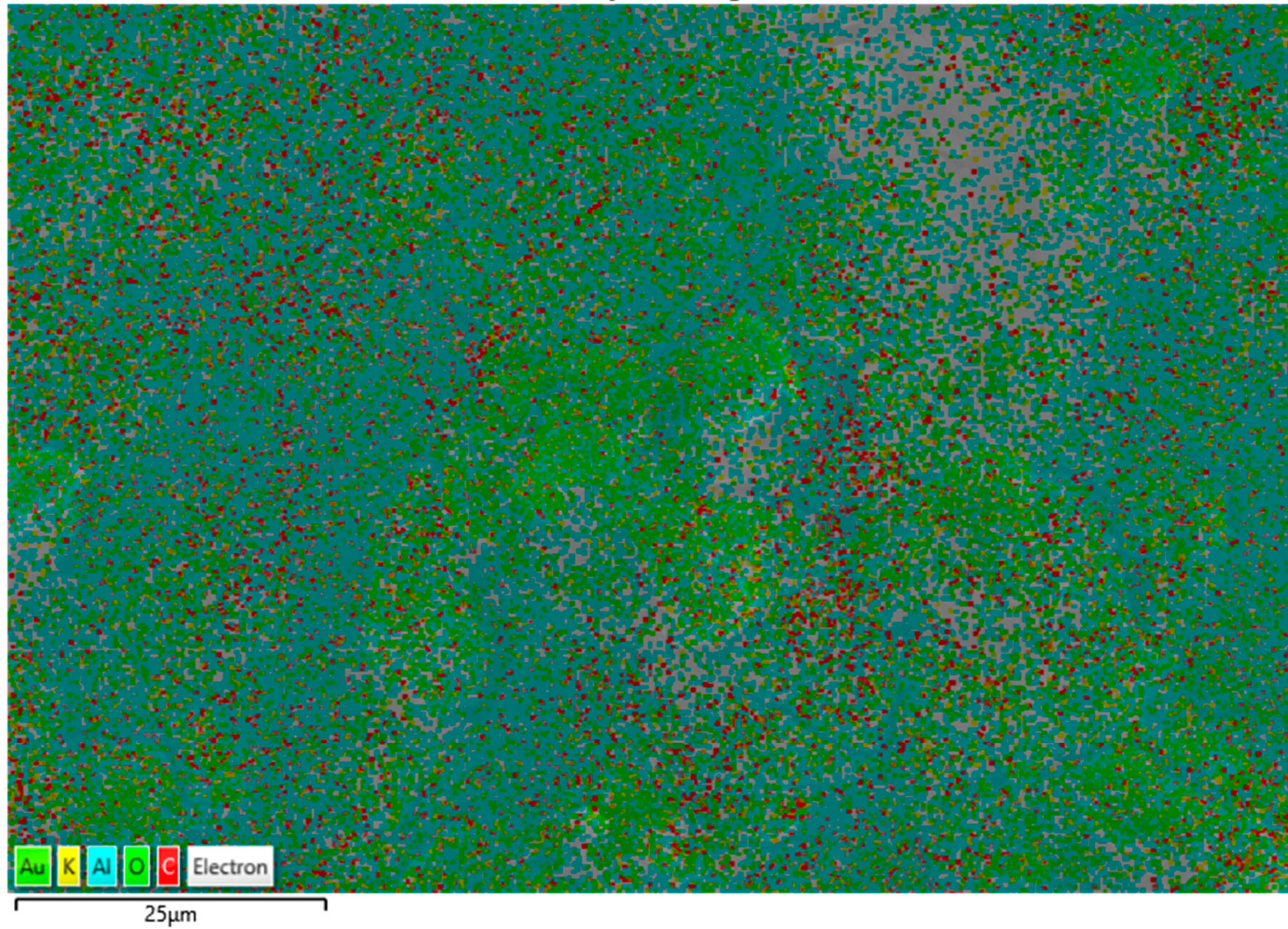

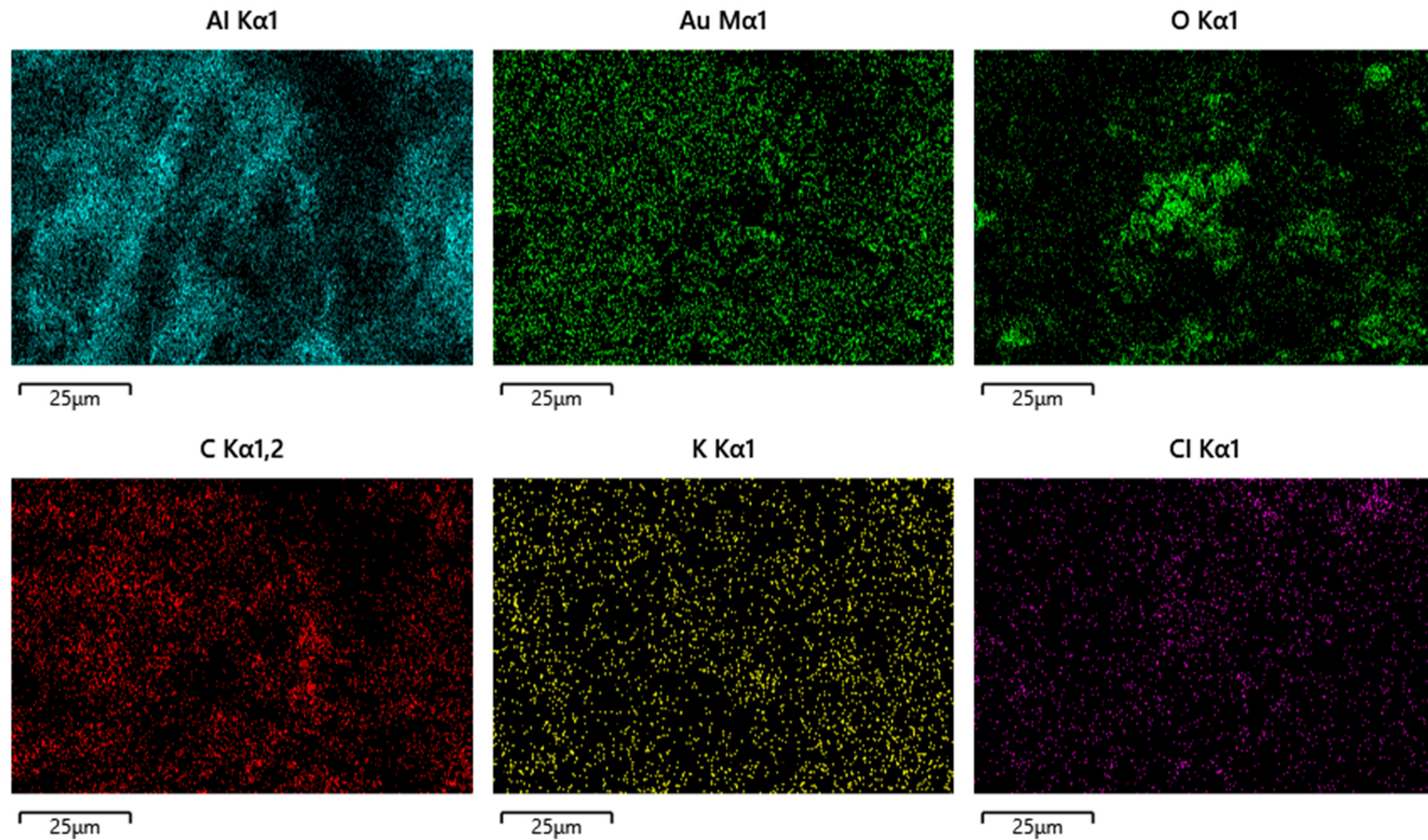

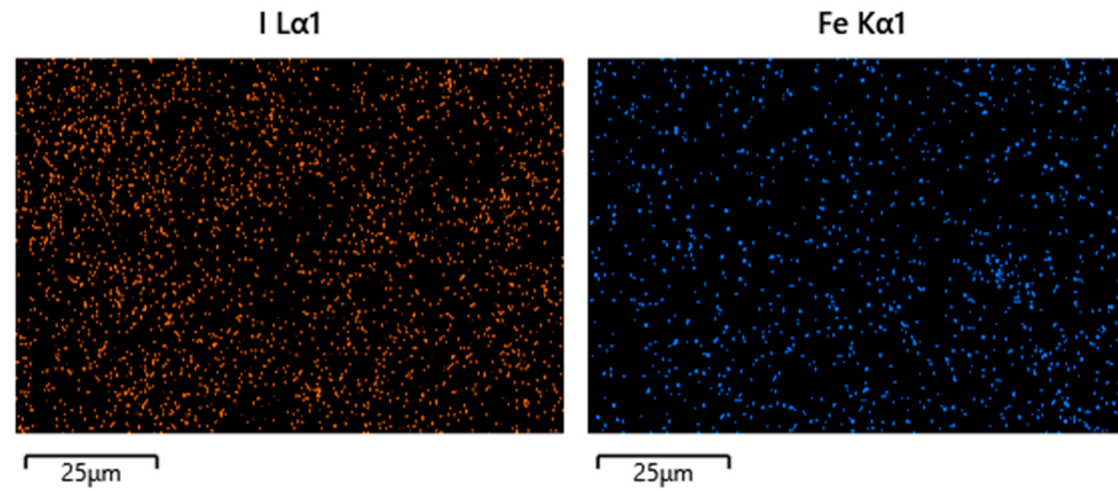

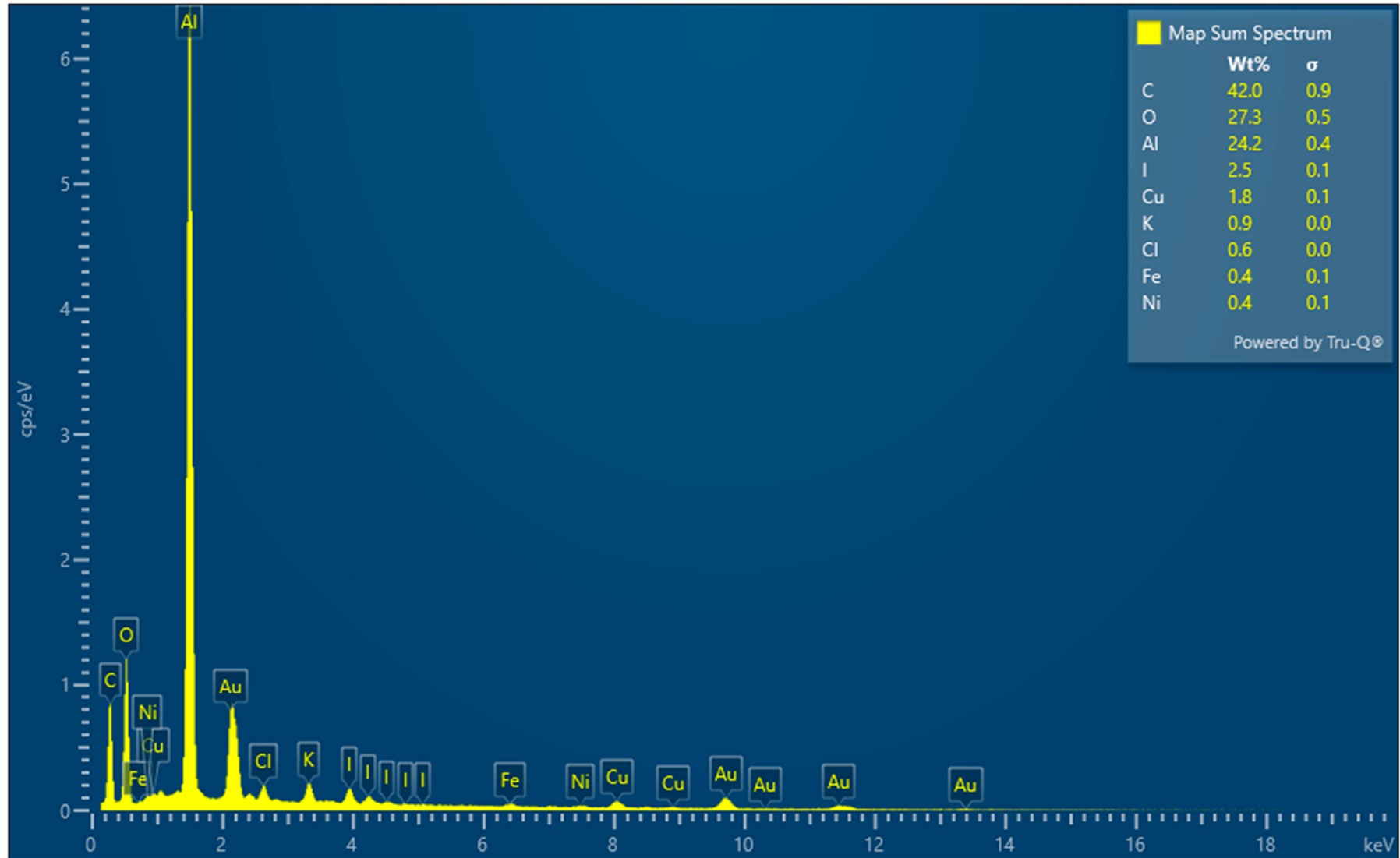

Supplement: Supplementary file 1 [file ijms-25-04949-s001.zip › S1-EDS AVPVPThymol-I2.pdf]

EDS Layered Image 9

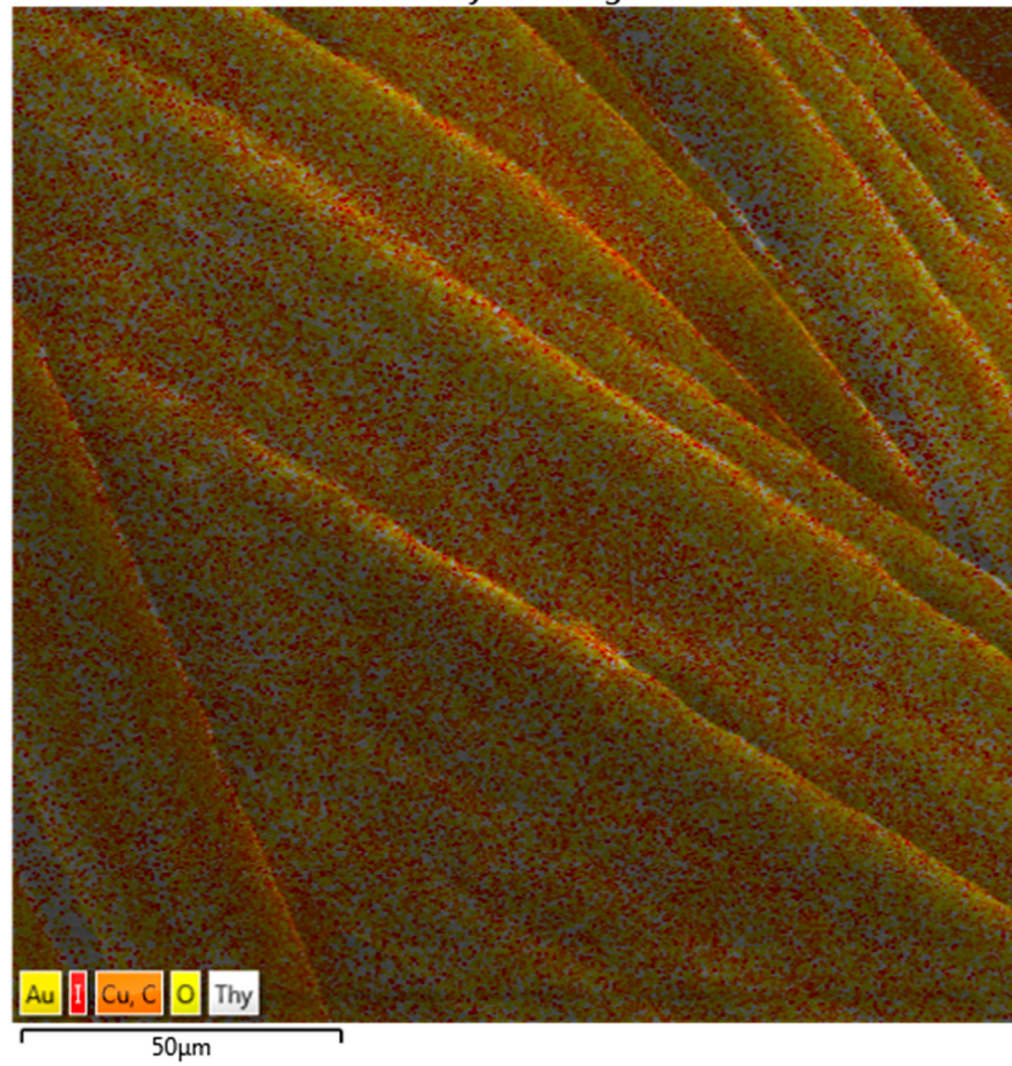

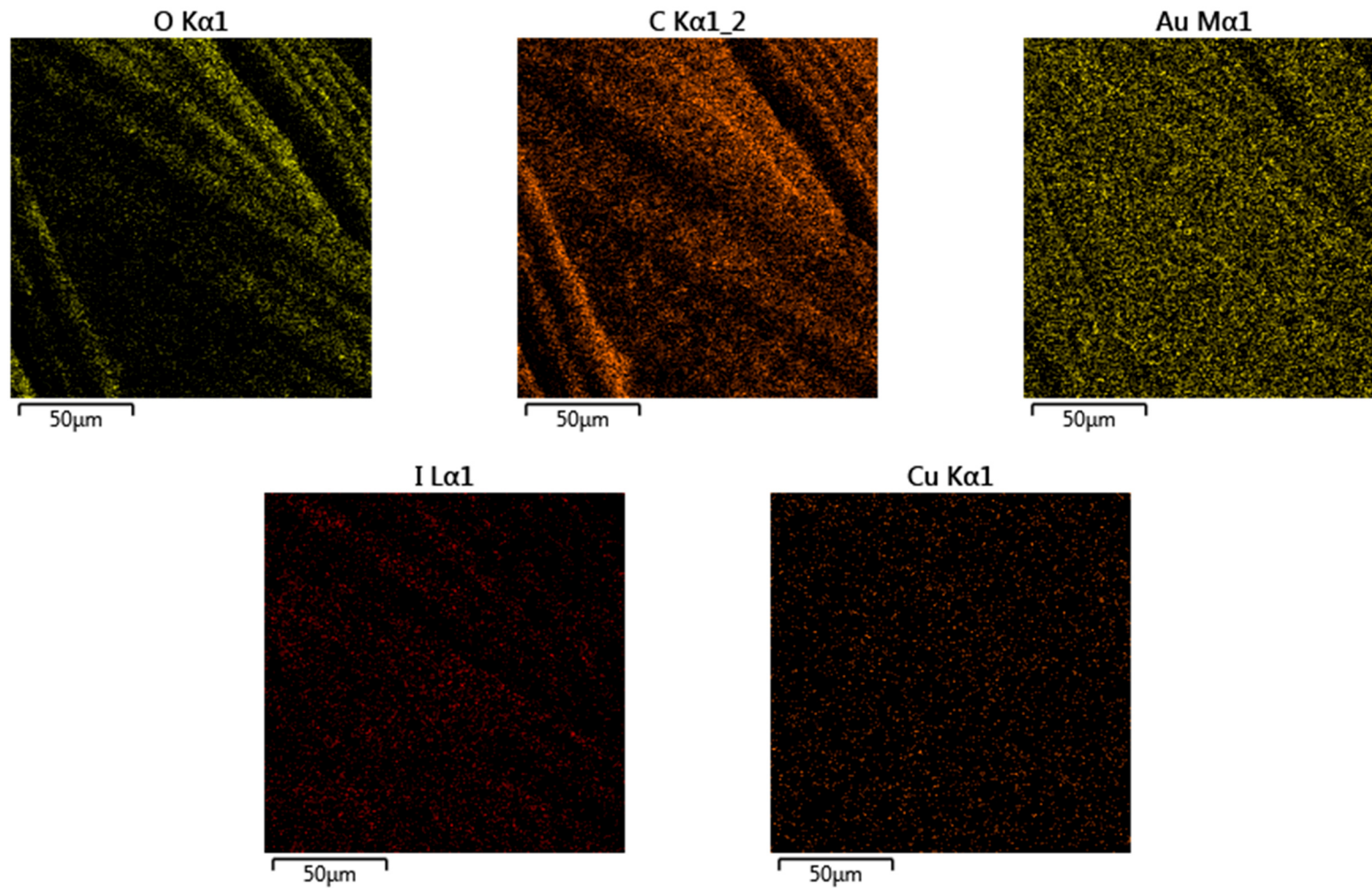

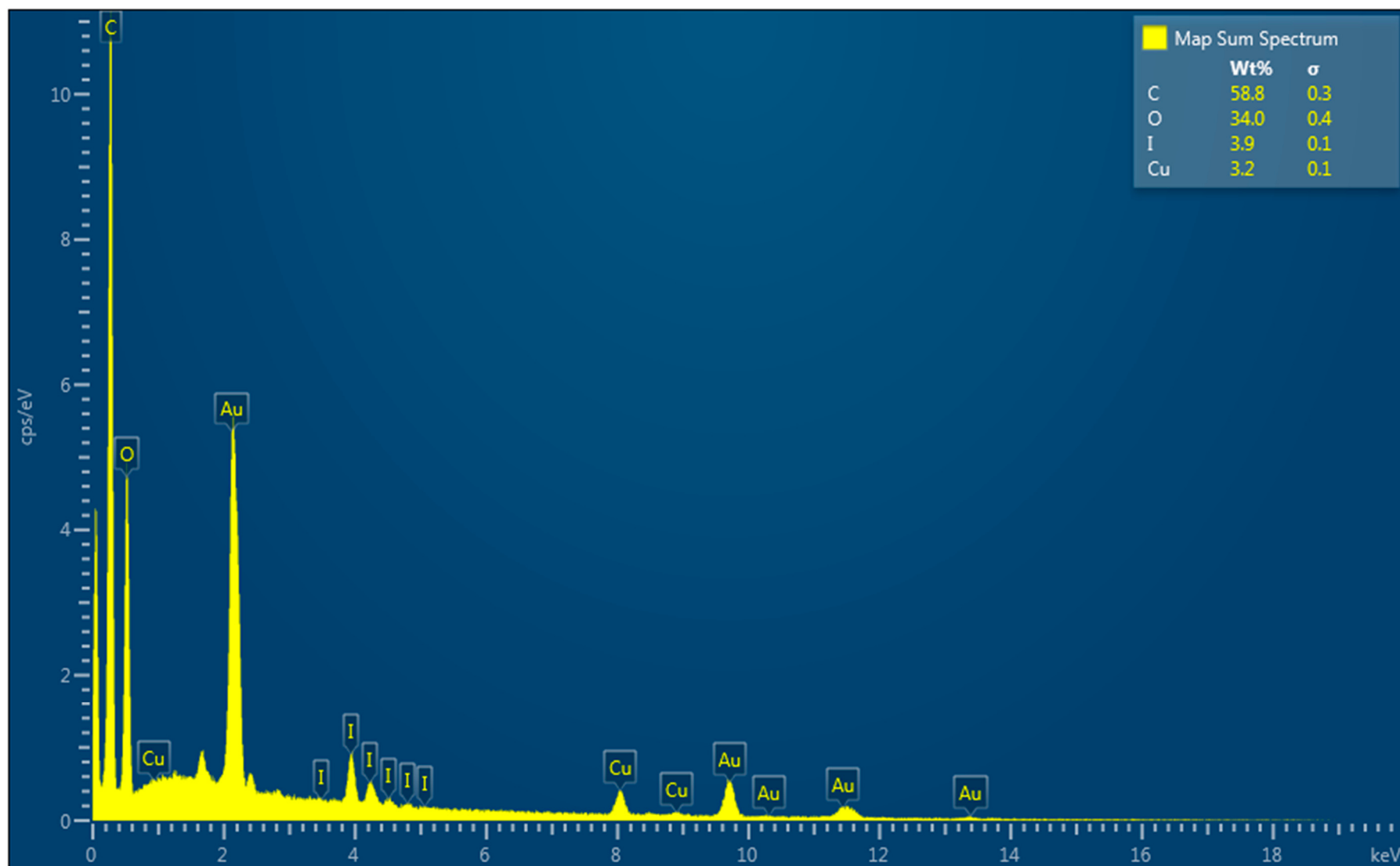

Supplement: Supplementary file 1 [file ijms-25-04949-s001.zip › S2-EDS AVPVPThymol-I2-Suture.pdf]

EDS Layered Image 5

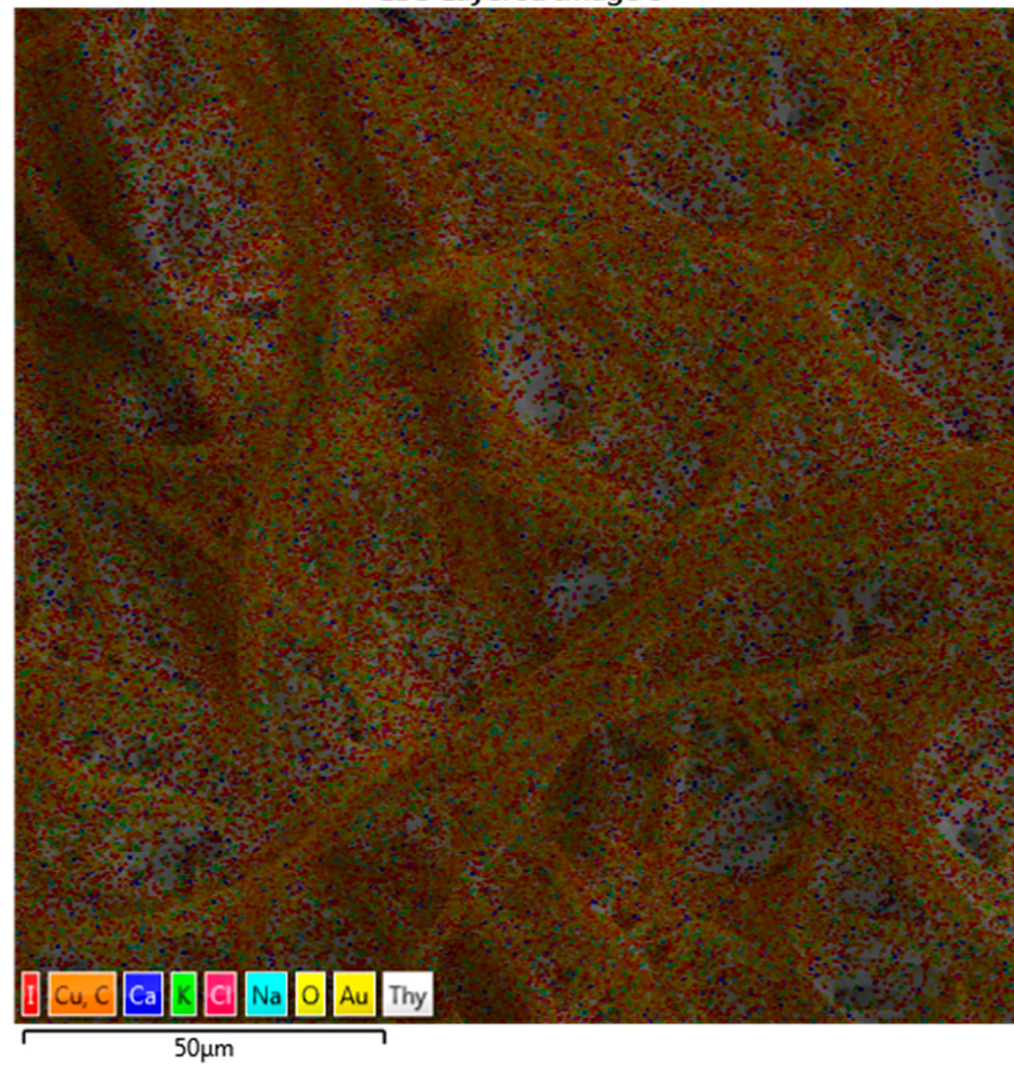

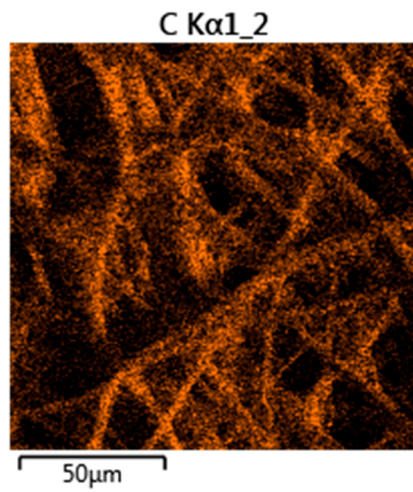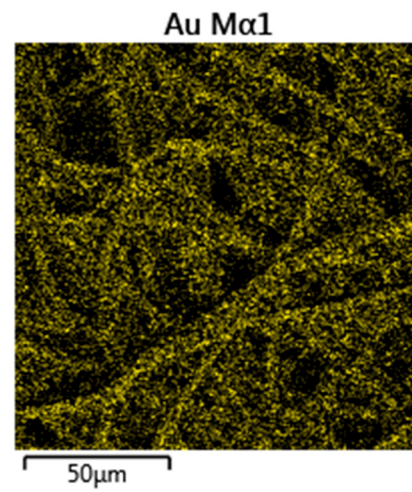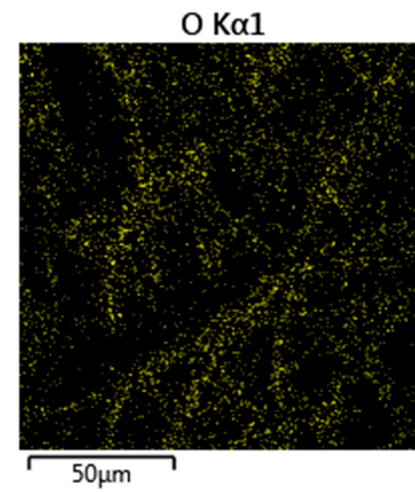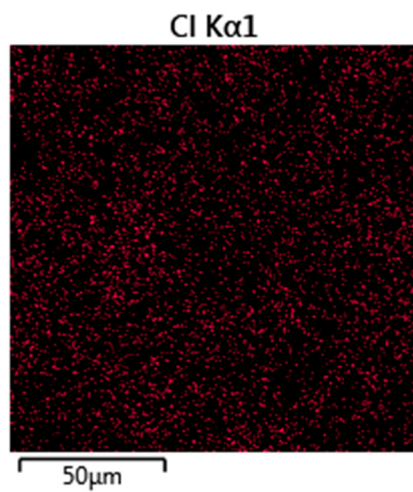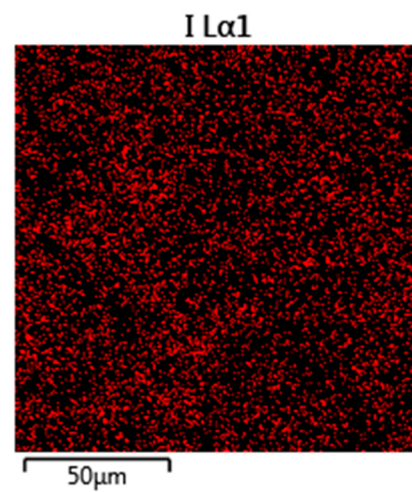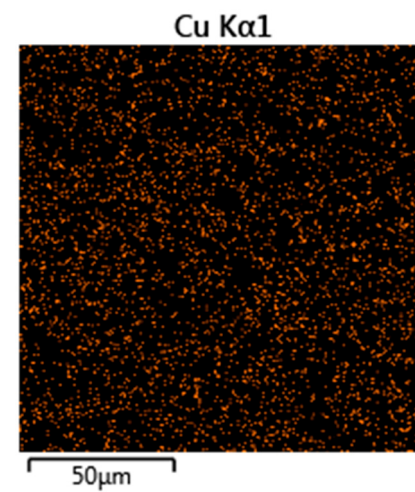

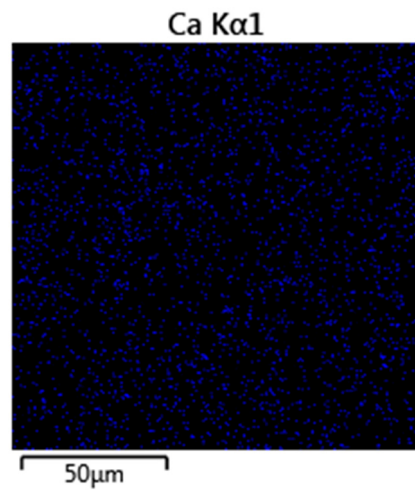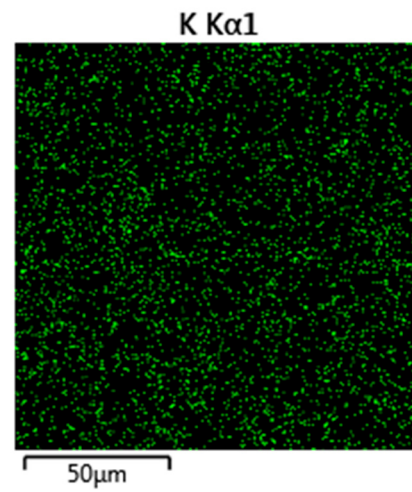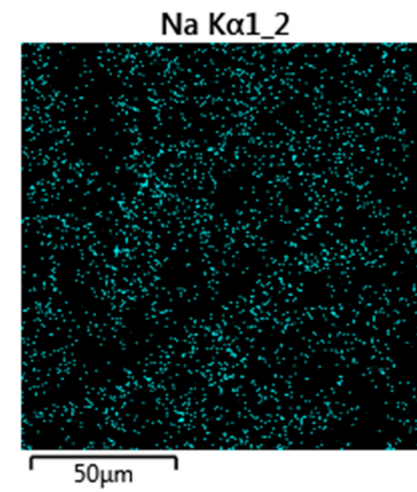

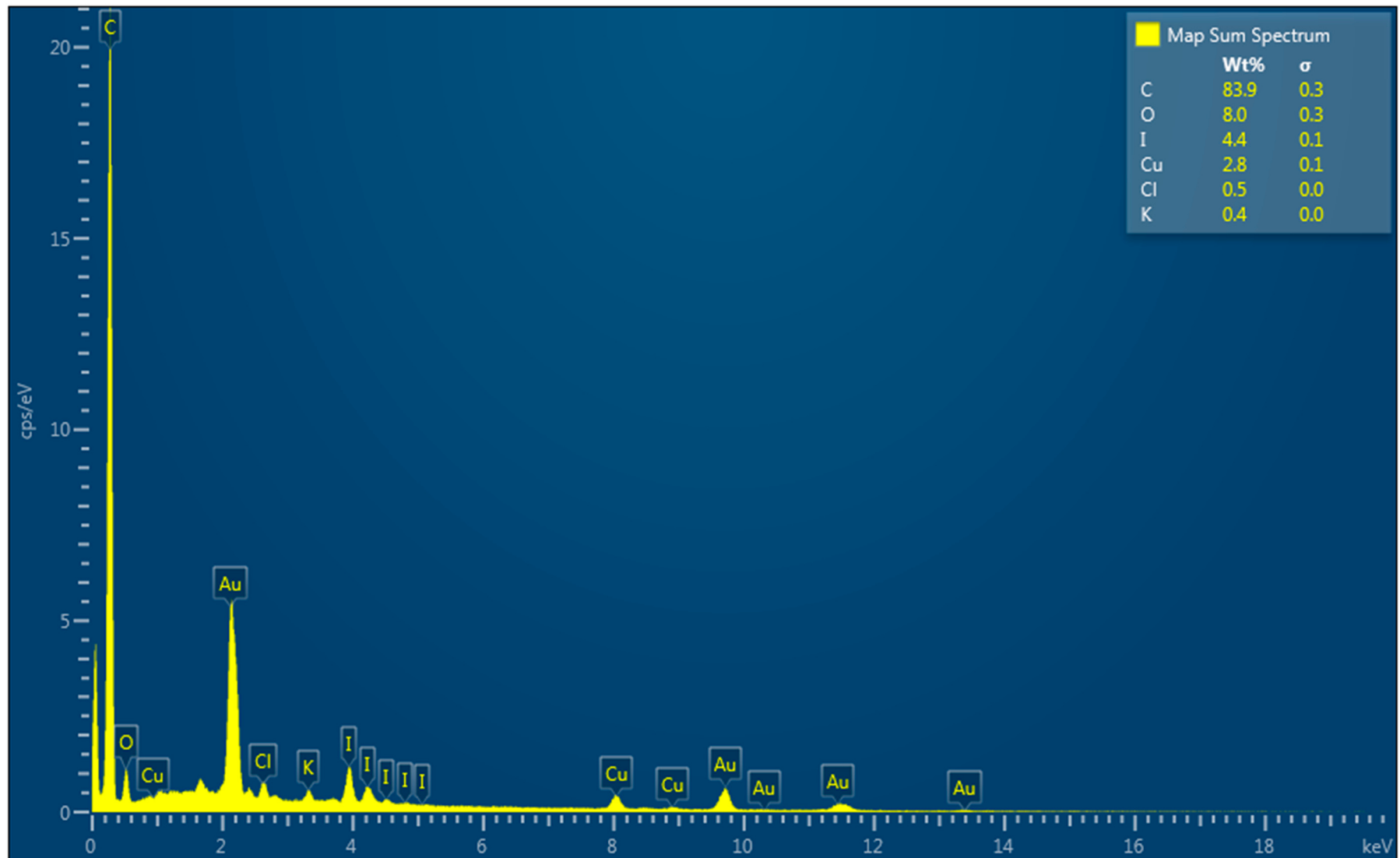

Supplement: Supplementary file 1 [file ijms-25-04949-s001.zip › S3-EDS AVPVPThymol-I2-Facemask-white dense.pdf]

EDS Layered Image 7

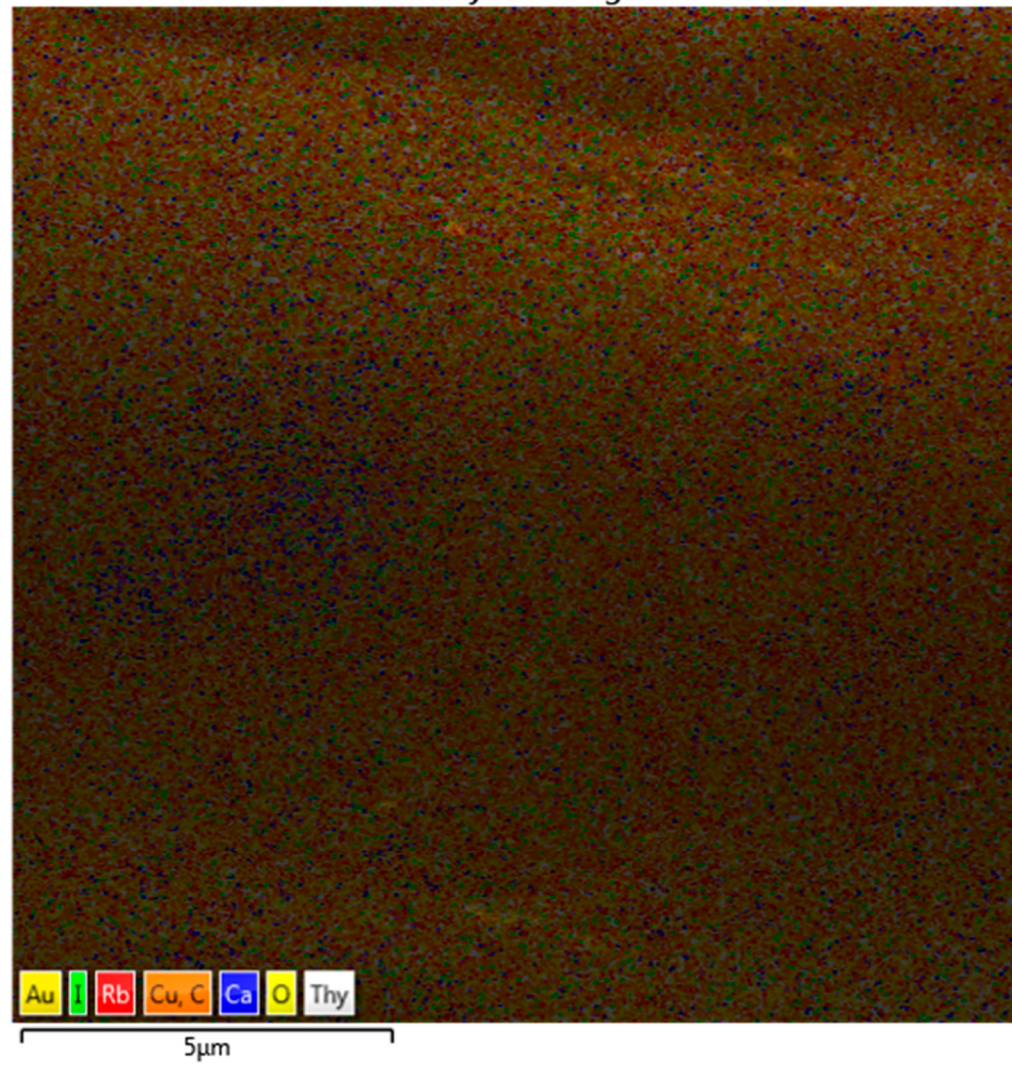

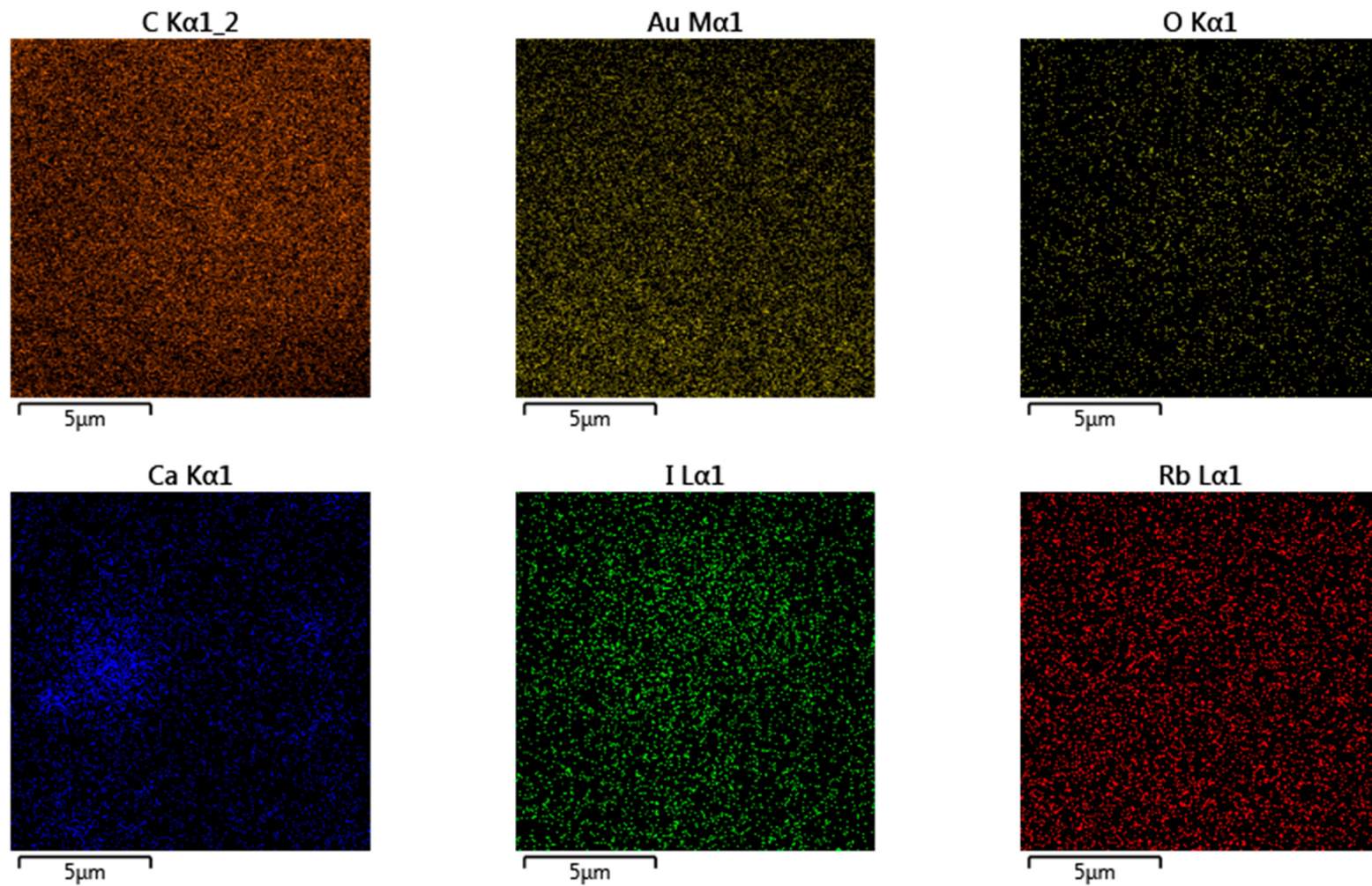

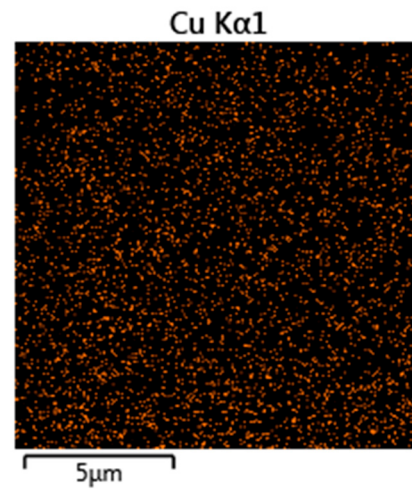

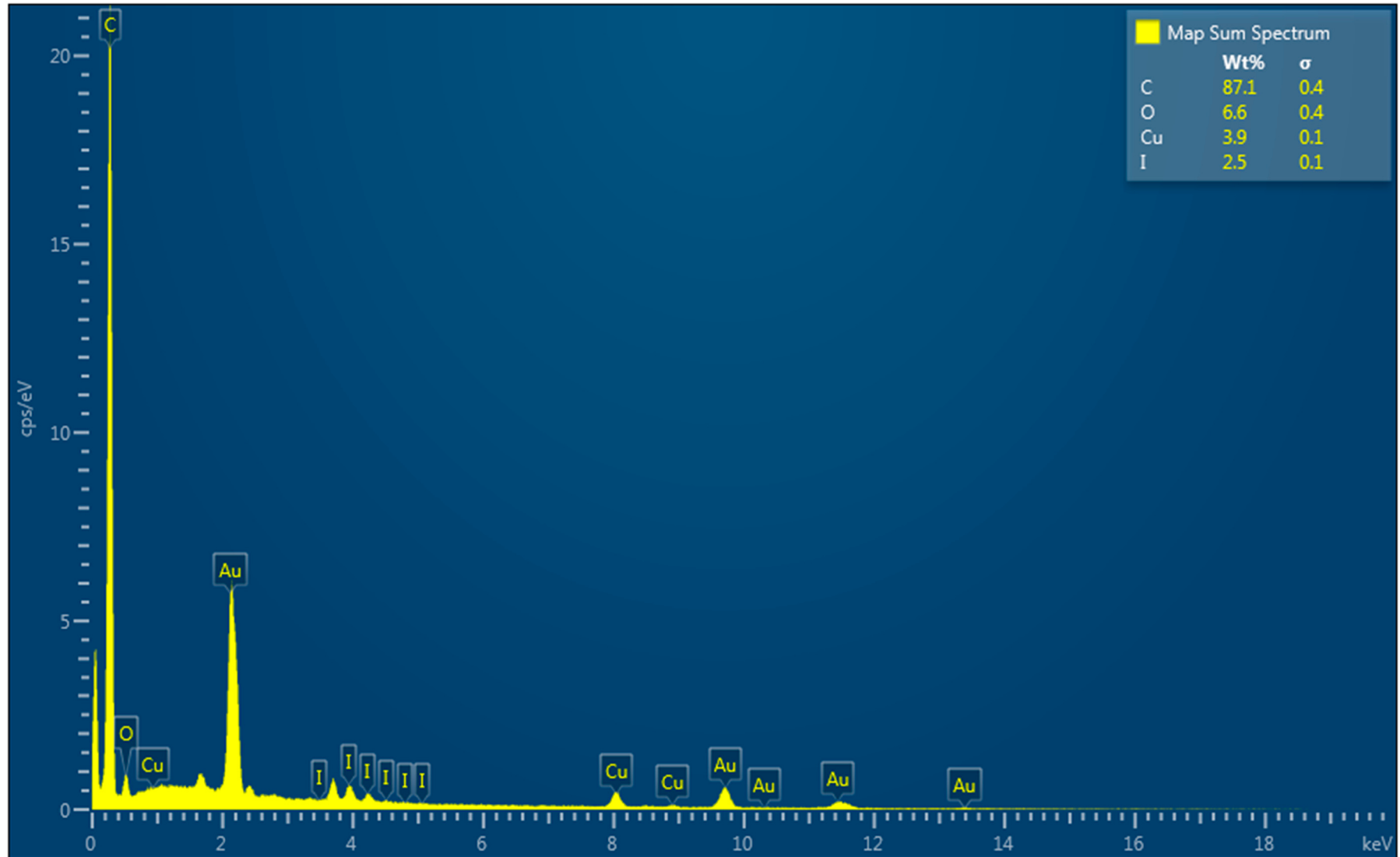

Supplement: Supplementary file 1 [file ijms-25-04949-s001.zip › S4-EDS AVPVPThymol-I2-Facemask-blue net.pdf]
